# Using a comfort zone model and daily life situations to develop entrepreneurial competencies and an entrepreneurial mindset

**DOI:** 10.3389/fpsyg.2023.1136707

**Published:** 2023-05-15

**Authors:** Marco Van Gelderen

**Affiliations:** Department of Management and Organization, Vrije Universiteit Amsterdam, Amsterdam, Netherlands

**Keywords:** entrepreneurial mindset, entrepreneurial competencies, enterprising competencies, entrepreneurship education, experiential education, surprise

## Abstract

This article presents a novel experiential learning format that aims to develop participants' entrepreneurial competencies and entrepreneurial mindset. Furthermore, this study investigates factors that promote individuals' competency development and mindset formation when using this learning format. In this format, students practice enterprising behavior in daily life, rather than by starting a venture. Teams of participants receive a set of eight to 10 challenges. Each challenge asks participants to create value for other people. The challenges are not revealed until the exercise starts, and they are worked on for 1 or 2 days full-time. Each challenge allows participants to practice the competencies of generating ideas for opportunities, taking action, perseverance, networking and network utilization, teamwork, and convincing others. Collectively, this contributes to developing an enterprising mindset. This format is based on a comfort zone model and aims to promote significant learning in a short time. After a week, each participant submits a reflection on their actions during the experiential part. In this study, we analyze the experiences of 198 participating students from six courses in five countries to bring out the factors that contribute to students staying in versus leaving their comfort zone, and the types of learning which result. Learning occurs when participants leave their comfort zone and have experiences that surprise them, leading to novel realizations. Key to learning is the element of surprise.

“*A pessimist sees the difficulty in every opportunity*

*An optimist sees the opportunity in every difficulty”*

–Winston Churchill

“*Fortes fortuna adiuvat*

*Fortune favours the bold”*

–Terentius

## Introduction

Governments and organizations worldwide have placed great importance on fostering entrepreneurial competencies and an entrepreneurial mindset among students, for example, the European Union (Bacigalupo et al., 2016) and the OECD (2022). This emphasis on entrepreneurial behavior extends beyond start-ups and business sectors and acknowledges the potential for value creation beyond economic gains (Lindberg et al., 2017b). For example, the EU EntreComp framework (Bacigalupo et al., 2016) takes a broad view of entrepreneurship and defines it as “the act of identifying opportunities and ideas and transforming them into value for others, whether financial, cultural, or social[sic]” (p. 16). The call for papers for this Frontiers special issue echoes this broad view. It posits that an entrepreneurial mindset is increasingly crucial for individuals to develop sustainable careers across diverse societal sectors, as changing labor relations require employees to possess entrepreneurial competencies. Conversely, organizations rely on their employees' innovative and intrapreneurial activities for business survival, thus increasingly expecting all levels of employees to engage in entrepreneurial activities. The call for papers invites research into the development of individuals' entrepreneurial mindsets that are directed toward the creation of value in various forms, not limited to economic outcomes.

This article responds to this call and has two objectives. First, it aims to contribute to the knowledge on the development of entrepreneurial competencies and mindsets by presenting a novel experiential learning format, which the interested entrepreneurship educators may potentially want to adopt. The format's detailed description adds to the body of work on pedagogical interventions (cf. Daniel, 2016; Lindberg et al., 2017a; Hultén and Tumunbayarova, 2020). The format is specifically designed to be relevant for both business- and non-business students. It is geared toward populations of so-called “pre-experience” students, the vast majority of whom neither own a business nor necessarily aspire to start or run one in the future. The novelty of the learning format lies in decoupling entrepreneurial behavior from starting to running a business. Instead, participants make use of daily life opportunities to create value for others, where value can be of any kind, not necessarily economical. As advocated by leading entrepreneurship educators (Lackéus, 2015; Hägg and Gabrielsson, 2019; Hägg and Kurczewska, 2022), the learning format is experiential, does not only concern ideation but also execution, and creates actual value for others external to the class. After presenting the learning innovation in detail, it discusses its pedagogical basis: the comfort zone model. The choice to utilize the comfort zone model is motivated by its potential to challenge habitual behaviors and facilitate rapid and substantial learning.

The second objective of this article is to uncover factors that promote individuals' competency development and mindset formation when using this format. To this end, this study uses reflection reports generated by participants and provides evidence for which types of learning occur, when such learning arises. The findings are derived using a qualitative content analysis of submitted individual reflection reports by 198 participating students from six courses in five countries. The study finds that learning arises when there is an element of surprise involved. Adaptation of current beliefs occurs when participants leave their comfort zone and have experiences that surprise them. These findings represent a second contribution, next to the description of the learning format, to the knowledge of the development of entrepreneurial competencies and mindsets.

This study proceeds as follows: first, it lays the groundwork by discussing competencies and mindsets in the context of entrepreneurship. This is followed by a discussion of the comfort zone pedagogical model. Then, the learning format is outlined in detail. Moving to the empirical research questions, the method section explains the data and research procedures. The findings section reveals what factors contribute to staying in the comfort zone vs. venturing outside of it, and the types of realizations that occur when participants learn from their experiences. This study concludes by listing the strengths and limitations of the presented learning format and by outlining future research possibilities.

## Entrepreneurship, competencies, and mindset

In its narrow conceptualization, entrepreneurship is about venture creation, self-employment, business development, product/service innovation, and growth. In its wide conceptualization, entrepreneurship is about life competencies that can be applied in any setting, such as autonomy, creativity, and taking initiative and risks, all in the context of value creation (Lackéus, 2015). When subscribing to the broad conceptualization of entrepreneurship, the term “enterprising” is often used, as enterprising does not imply venture creation. Value creation can take a multitude of forms and shapes, of which venture creation is only one. The learning format described in the next section is aimed at the development of enterprising competencies and an enterprising mindset. However, given that the term entrepreneurial is more current than the term enterprising, the term entrepreneurial will be used in the remainder of this article. The discussion revisits the distinction between enterprising and entrepreneurial.

A sizeable percentage of experiential formats in entrepreneurship education is focused on developing entrepreneurial competencies (Lackéus, 2015; Lilleväli and Täks, 2017; Nabi et al., 2017). Competencies concern the combined and integrated components of knowledge, skills, and attitudes (Van Gelderen, 2020). Applied to entrepreneurial behavior, entrepreneurial competencies are knowledge, skills, and attitudes directed to taking entrepreneurial actions and creating value for others and society, whether the value that is created is financial, cultural, or social (Gibb, 1993; Bacigalupo et al., 2016; Lilleväli and Täks, 2017). Competencies are not fixed traits, they can be developed and learned through experience and training (Kyndt and Baert, 2014; Lackéus, 2020). The same goes for mindset (Lindberg et al., 2017a,b; Hultén and Tumunbayarova, 2020). The entrepreneurial mindset, the focus of this special issue, is a term that has been used in a variety of manners in entrepreneurship literature. Recently, multiple authors have made efforts at conceptual clarification (Naumann, 2017; Daspit et al., 2021; Kuratko et al., 2021). The number of conceptualizations of the entrepreneurial mindset is so large that it allowed Daspit et al. (2021) to conduct a qualitative content analysis. Based on the commonalities in 61 articles, they proposed the following definition: “Entrepreneurial mindset is defined as a cognitive perspective that enables an individual to create value by recognizing and acting on opportunities, making decisions with limited information, and remaining adaptable and resilient in conditions that are often uncertain and complex” (Daspit et al., 2021, p. 6).

There are similarities between this definition and EntreComp's broad definition of entrepreneurship referred to in the introduction section (Bacigalupo et al., 2016). The entrepreneurial mindset is focused on value creation and concerns not only ideation but also action. Attitude, skills, and knowledge are all involved in creating value by recognizing and acting on opportunities under specific conditions such as complexity, uncertainty, and limited information. Daspit et al. (2021)'s definition of entrepreneurial mindset as a cognitive perspective has reference to the attitudinal component, in line with Merriam Webster's definition of mindset as a mental attitude or inclination, and the Oxford online dictionary's definition of mindset as an “established set of attitudes.” We will assume that those with an entrepreneurial mindset require entrepreneurial competencies and that the development of entrepreneurial competencies in turn helps to develop an entrepreneurial mindset.

Before presenting the learning innovation in detail, the article will first discuss its pedagogical basis: the comfort zone model. Current theories of competency development suggest that once a satisfactory level of performance is attained, actions become automatic and processed without explicit conscious control (Keith et al., 2016). As a result, performance plateaus. As Baron and Henry (2010, p. 51) assert, “In many different domains, individuals experience swift improvement in their performance until they reach a level that is perceived as satisfactory. They then encounter a plateau and do not make any further progress. Consequently, many individuals remain at a certain level of proficiency for years or even decades, despite accumulating substantial experience as measured by their active engagement in a given domain.” This poses a significant challenge to competency development. The comfort zone pedagogical model helps to disrupt routine behaviors and can promote significant learning in a short time.

## The underlying pedagogy: learning outside the comfort zone

**Figure 1:**
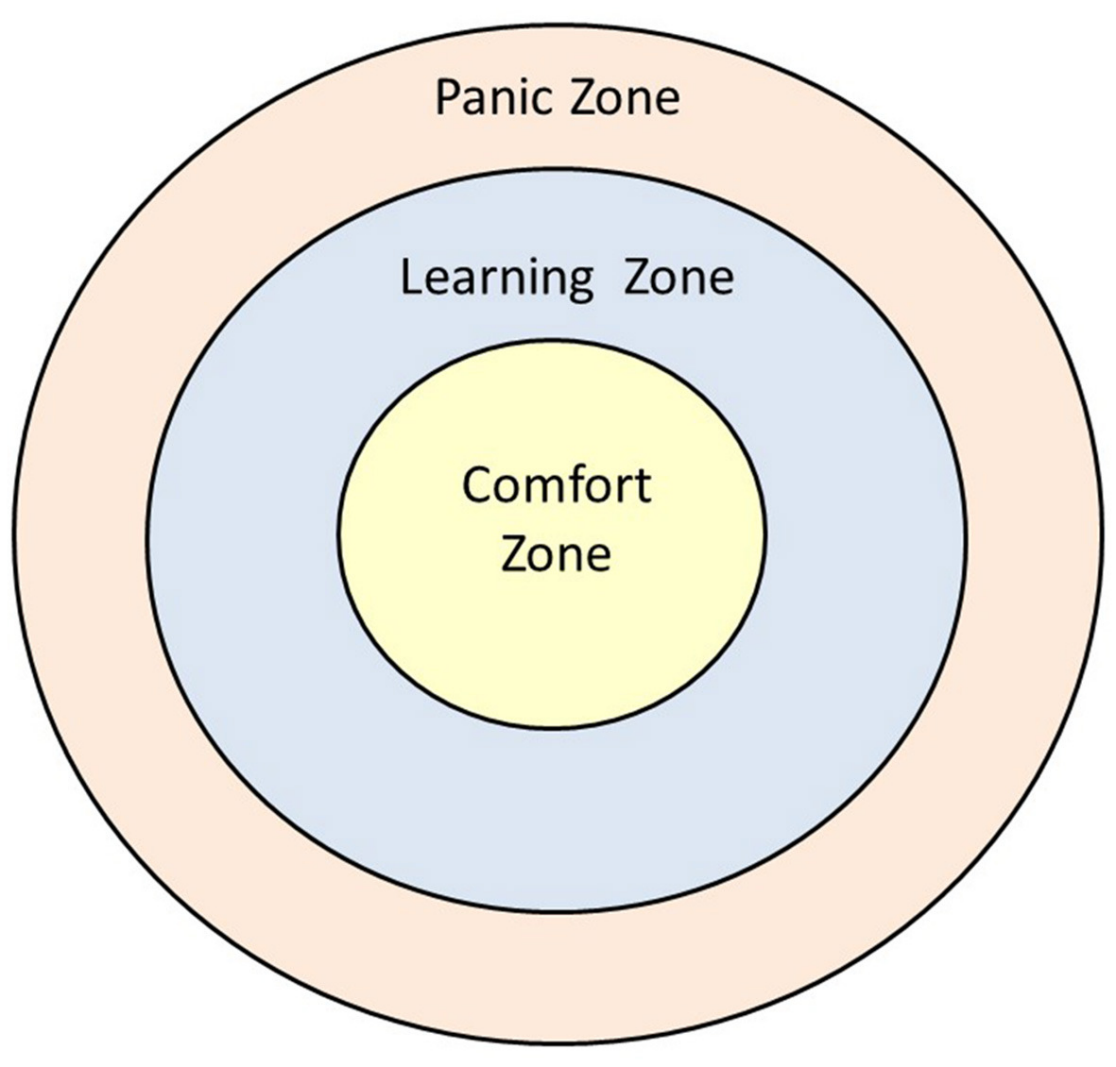
Comfort zone model showing comfort, learning, and panic zones. Source: Adapted from Panicucci (2007).

The term comfort zone was originally used in the context of warm clothing but started to refer to a learning model in the 1990s, particularly in the literature regarding outdoor adventure education (Nadler, 1995). In outdoor adventure education, participants engage in activities that seem to contain a high degree of physical risks, such as mountain climbing. In reality, the facilitator has managed the risks to the extent that no serious physical harm should occur (Priest and Gass, 2005; Brown and Fraser, 2009). By surpassing themselves and acting despite fear, or even overcoming their fear, participants are considered to increase their perceived level of competence regarding the activity (for example, “I am able to climb the mountain”). This is then thought to generalize to life domains outside of the realm of outdoor adventure activities (Priest and Gass, 2005) by changing self-beliefs (for example, “I am a person who is able to overcome challenges and surpass myself”). In the learning format presented in this study, the risks are psychological and social, rather than physical [although physical outdoor training has also been shown to improve entrepreneurial competencies (Padilla-Meléndez et al., 2014)]. This makes it possible to learn from failure—one can learn from a failed attempt at convincing someone, for example, whereas there may be little left to learn when physically falling off a cliff. Nevertheless, the comfort zone learning model (Figure 1) equally applies.

The inner circle of Figure 1 depicts the comfort zone. The comfort zone, in the psychological sense, is a metaphor expressing that there are behavioral patterns that are safe, known, familiar, predictable, secure, comfortable, and reliant on routines, in which people feel competent and have minimal anxiety. Outside of the comfort zone are behaviors that move people into territory that is unknown, unfamiliar, uncomfortable, unpredictable, unexpected, and risky (Nadler, 1995). In such conditions, participants may enter something that has been referred to as a learning, stretch, or groan/growth zone, where they struggle and can attempt to learn new behavioral patterns. When newly mastered behaviors become comfortable and routinized, the comfort zone is expanded. It is also possible for a participant, metaphorically speaking, to bypass the learning zone and panic, thus entering the panic zone, which is then followed by a retreat to the comfort zone. From an educator's perspective, the challenge is to create exercises that stimulate students to leave their comfort zone and enter the learning zone, without being so overwhelming that they enter the panic zone and retreat into the comfort zone.

Priest (1993) classified the movements between the comfort and out-of-comfort zones as a function of risk and competence. As a person gains experience in an outdoor adventure activity, their perceptions of risk and competence are thought to change. Both risk and competence can initially be underestimated or overestimated. By leaving the comfort zone, perceptions of risk and competence can become more astute. If successful in the activity, perceived competence grows, and the perceived risk of threat or harm lowers (and/or the perceived ability to manage the risks increases). The comfort zone has expanded, and the person can seek new challenges, ready to enter the learning zone again. For learning to occur in a comfort zone model, the experience should initially be uncomfortable, but the teacher and the educational setting, including the support offered by fellow student team members, should provide psychological safety.

### The comfort zone: stay in or venture out?

As stated earlier, in the comfort zone, there is low arousal, low stress, low anxiety, familiarity, and routine. Moreover, the comfort zone functions as a security mechanism that minimizes harm. By remaining in their comfort zone, people can avoid risk and uncertainty and thus avoid situations or activities that may prove dangerous. Furthermore, most activities in the comfort zone are done automatically (Bargh and Chartrand, 1999; Kahneman, 2011), freeing-up cognitive space and providing an uninterrupted level of comfort. Why would someone be willing to leave this zone?

Various motivation theories provide an answer to this question. Examples of theories positing that humans are motivated to master new tasks and skills are the Self-Determination Theory (Deci and Ryan, 2000); Maslow's hierarchy of needs, particularly the need to actualize one's potential (Maslow, 1943); and competence or mastery motivation (White, 1959). Other authors float the idea of humans being motivated to seek optimal challenge, as featured in Csikszentmihalyi's theory of flow (Csikszentmihalyi, 1990) and Pink's notion of productive discomfort (Pink, 2011). However, seeking to be challenged does not only need to be explained from a growth or mastery motive. Theories of thrill and sensation-seeking (Zuckerman, 1979) suggest that some people are motivated to do so just for the thrill it provides. While all these theories suggest that people may be motivated to leave their comfort zones, anecdotal evidence suggests that only a few people habitually challenge themselves and that most people remain within their comfort zone for most of their time. Thus, a learning format using a comfort zone model needs to be designed in such a way that it pushes participants out of their comfort zone. By providing first-hand experiences that allow them to break habits and routines, participants are challenged to be resilient and use their coping skills and encounter unexpected lessons.

### The learning zone: changed beliefs and expansion

What happens once in the learning zone? Various learning theories provide explanations for the learning effects that occur when people are pushed outside of their comfort zone. As suggested earlier, habitual and automatized behaviors (Bargh and Chartrand, 1999) are challenged in the learning zone. When experiences disconfirm originally held beliefs, various theories can explain what happens (Brown, 2008). One is Piaget's theory (Piaget, 1980), which states that new experiences can result in assimilation and accommodation. The question is, what happens if a new lesson is learned? Here, generalization or transfer is key. With assimilation, new experiences are integrated into one's existing belief systems. If the experience is interpreted as a local, one-off, disconnected lesson, not much changes in someone's belief system. With accommodation, on the other hand, current beliefs are adapted or newly formed. If a new generalized lesson is learned, then accommodation takes place. Partly based on Piaget's work, Kolb (1984) developed his well-known model of the experiential learning cycle, in which there are four stages in learning that follow each other. To start with, *concrete experience* is followed by *reflection on that experience* on a personal basis. This may then be followed by the derivation of general rules describing the experience or the application of known theories to it (*abstract conceptualization*) and, hence, to the construction of ways of modifying the next occurrence of the experience (*active experimentation*), leading to the next concrete experience. As Kolb's model shows, reflection is an essential ingredient in experiential learning. Sometimes learning does not occur directly but comes afterward by reflecting on one's experience. The next section describes how the comfort zone pedagogical model is operationalized in the learning format, which includes the use of individual reflection reports.

## Description of the learning format

The aim of the learning format is the development of entrepreneurial competencies. As such, the format is more geared toward creating value for others rather than appropriating value for oneself (the main appropriated value is learning). The challenges typically do not involve starting or running or operating a new business, but the creation of value for others in daily life. Each challenge facilitates the integrated practice of each of the six competencies covered in the format: generating ideas for opportunities, taking action, perseverance, networking and network utilization, teamwork, and convincing others. The competencies are jointly practiced because the competencies are interdependent (e.g., in the case of taking action, one takes action on ideas, one perseveres with actions taken, one takes actions to network, work together, and convince others) (RezaeiZadeh et al., 2017).

Teams of typically four participants receive a set of eight to 10 different challenges, which they work on for 1 or 2 days full-time.^1^ The challenges encourage participants to make use of opportunities to create value for others offered by everyday life. They ask participants to connect with people, help someone out, express curiosity, organize something, invite someone, take part, and share. One example is linking two (groups of) people (not yourself or fellow students in your class), who do not know each other before, meaningfully in a way that creates value for them both. Proof: Picture of these two (groups of) people. Within a 1- or 2-day period (both time frames are suitable), each of the challenges allows participants to create value for other people. During these “action learning days,” participants are expected to be available for the entire day(s) and to cancel any obligation they may have. Supplementary material provides a sample course or module outline showing the instructions to students.

The challenges pose significant novel experiences for many participants, or at least the possibility to engage in such. Teams are expected to work on not just one challenge but on all eight or 10, which must be tackled within 1 or 2 full-time days spent off-campus. Challenges can be combined or rephrased, provided that this would serve the practice of entrepreneurial behavior in daily life. Each challenge requires taking action, and as such invites participants to think on their feet and act on the go (rather than merely submit a written plan). It is important that the students venture out from the university, as unfamiliar surroundings contribute to leaving their comfort zone. Moreover, opportunities to create value in daily life are much more plentiful off-campus. The challenges are designed in such a way that they make use of daily life situations. As such, there is no financial cost to participants, and there is no financial barrier to taking part.

The format is team-based. Teams of four are announced just the day before. As the format aims to promote teamwork and networking, the instructor puts the teams together, rather than have participants form teams themselves. Four is an optimal number as it creates variety in the teams, without the team becoming so large that members can easily freeride. If international students are present, instructors are advised to divide them evenly over the teams so that each team has at least one member who speaks the local language and is familiar with the local culture. To promote autonomous, self-starting behavior, the instructor immediately leaves the teams to themselves after handing out the challenges (although it is highly recommended to list your phone number on the handout for emergencies).

The features of this learning format are expressions of the underlying pedagogy: the comfort zone model (see previous section for elaboration). The use of the comfort zone pedagogical model requires that the challenges be not revealed until the exercise starts. To facilitate out-of-comfort-zone experiences, the challenges are kept secret until they are handed out on the morning of the first off-campus day. The idea is that participants cannot prepare for the challenges. Also, public knowledge of the challenges is to be prevented, as it could reduce the out-of-comfort zone experience.^2^

After receiving the challenges, the student teams often feel thrown off-balance and feel that it will be hard to be entrepreneurial in the 1 or 2 days allowed and with the limited resources they have. However, as the previous section clarified, pushing participants out of their comfort allows for the learning of new behavioral repertoire. This is further enhanced by keeping the challenges secret until they are handed out on the morning of the first off-campus day. The idea is that participants cannot prepare for the actual challenges. Prior knowledge of the challenges is to be prevented, as it would reduce the out-of-comfort zone experience. Students are instructed that they cannot prepare for the event and that they should just come to a central meeting point, bringing their ID card and their phone with them (and possibly a car, depending on the availability of public transport).

Growth and learning may not necessarily occur exclusively at the time of going through an experience (Hägg and Kurczewska, 2021). It can also occur later when reflecting on one's actions. A first post-experience opportunity for reflection occurs on the day after the action days when students present their experiences and achievements to the class. By presenting their actions to other teams, participants can compare their experiences with those of the other teams. A week later, participants are asked to submit their reflection reports. This timing is chosen to provide them with time to reflect while their memory is still fresh. See Supplementary material for the sample module outline which includes a grading schedule. In the reflection report, participants briefly describe what happened during the challenges and then focus their analysis on their own individual behavior. The assignment asks participants to analyze why they behaved as they did and to analyze situational and personal influences on their behavior. They are also asked to analyze what aspects can be improved and to make a plan to improve their entrepreneurial competencies.

Optionally (the format does not require prerequisite knowledge), the field days can be preceded by one or more days of lectures on the competencies covered in the format.^3^ They describe theory and research about each of the competencies studied and practiced in the learning format and can serve as readings and as a basis for lectures. Participants can be asked to incorporate constructs and theories from these readings in their reflections. In this way, they can show their ability to apply the literature to their experiences, and their experiences to the literature. Students are invited to treat the literature critically. Incorporating readings into the reflections can help students to change their mental models regarding the various competencies, thus working on the knowledge component of competencies. The students conclude by writing a short plan with specific steps for the future development of the competencies involved. This plan can be taken up in a later part of the course or the program, if such a possibility exists. In my own course, the learning format is followed by deliberate practice of an identified point for improvement (Van Gelderen, 2022).

The first objective of this article was to share the learning format and its underlying pedagogical basis. Next, it focused on the empirical analysis aimed at achieving its second objective: to uncover factors that promote individuals' competency development and mindset formation when using this format.

## Methods

### Sample

In total, 721 reflections from 24 courses given in the period 2013–2019 were available. To prevent saturation, reflections were selected which belonged to the first time a module was provided in a particular location (six locations in total as in Finland the module was provided at two different universities). The sample consisted of 198 individual reflections of participating students in five countries: Austria [in 2 different programs (20 + 40)], Finland [at 2 different universities (13 + 59)], Germany (6), the Netherlands (36), and Russia (29). The courses were offered at the university's bachelor's and master's levels. The sample consists of 55% male and 45% female students. Nearly all participants were between 20 and 24 years. In all cases, the course attracted students who voluntarily choose the course. It was never part of a mandatory program. Nearly all courses presented a mix of domestic and international students.

### Analytical approach

This study makes use of the reflection reports, in which students individually analyzed their behavior and the reasons for behaving as they did (for information on the assignment, see the Description of the Learning Format section as well as Supplementary material). All studied reflections are in English. The reflections were searched for the term listed in Table 1. The term comfort zone was neither used in the course readings nor was the concept frontloaded (in other words, it was not discussed in lectures preceding the experiential learning format). The term would only be discussed in class if it would come up during the presentations held afterward (see Description of the Learning Format section).

**Table 1:** Keywords and number of occurrences (in 198 reports).

**Keywords**					
Comfort	96	Fear	57	Learn	176
Comfort zone	52	Risk	105	Grow	38
Dare	14	Afraid	47	Realize	149
Courage	54			Surprise	56

The pieces of text brought up by the students in relation to the keywords were coded by the author using thematic analysis, as outlined by Braun and Clarke (2006) and Guest et al. (2012). Thematic analysis is a qualitative technique for identifying, analyzing, and reporting patterns (themes) in data. It does not involve counting phrases or words as is done in content analysis. In the first step, any factor related to the keywords in Table 1 is provided a code. These first-order codes are always literal to the text and do not involve an interpretation or evaluation. They concern the basic elements of raw data. Then, based on similarity, the first-order codes are grouped at a higher level of abstraction, and this process is repeated until a limited number of higher-order codes emerge, which are then labeled themes Guest et al. (2012). There was no second coder, partially out of privacy considerations as much of the information in the reflection reports is quite personal. This is a limitation of the research.

The analysis relies on the student's reflections. The reflections give insight into the student's experience and if, how, and why the exercise led to changes in their attitude, knowledge, or skills. One caveat regarding the reflections concerns the extent to which they are honest. After all, students were graded based on these reflections. However, as the marking schedule in Supplementary material shows, students were foremost graded on the depth of effort made when engaging with the challenges, the depth of the reflection, and the depth of application of the literature. Students were not graded on espoused competency proficiency. The instruction in the course guides explicitly states “The assignment is not meant to convince the lecturer that your enterprising skills are well-developed! It is not meant as proof that you were very enterprising. Rather, it should show that you are able to analyze your own behavior [sic], and that you are able to think of ways to improve.”

## Findings

### The comfort zone: stay in or venture out?

The first empirical research objective is to establish the factors that contribute to participants staying in or venturing out of their comfort zone, thus entering the learning zone. The thematic analyses reveal four themes (see Table 2). The first three are motivation, capability, and social norms. These themes correspond to the components of the Theory of Planned Behavior (Ajzen, 1991). The fourth theme relates to some attributes and experiences specific to the course design. For each theme, Table 2 provides first-order codes and illustrative quotes of factors relevant to staying in the comfort zone (marked “–”) and of factors contributing to leaving the comfort zone (marked “+”). The pressures to stay in the comfort zone may or may not be overcome. The factors marked “+” each provides antidotes to the pressures marked “–” in Table 2. Most of the factors listed in Table 2 marked “–” have a symmetrical counterbalancing factor marked “+.” For example, if a perceived lack of created value causes participants to stay within their comfort zone, the solution is to create more value; with team difficulties, the solution is to address them so that the teamwork can be improved. Because of this symmetry, the findings will be discussed per overarching theme (motivation, capability, social norms, and course-specific attributes).

**Table 2:** Staying in vs. leaving the comfort zone.

**Representative quotations**	**First order codes –: staying in CZ** **First order codes +: leaving CZ**	**Themes**
Looking closer at why this happens, I think the underlying process is that I can feel considerable fear of failure/rejection and value too highly what relevant others might think of me.	Fear of failure –
I am usually very nervous when faced a task containing many unknown factors and indeterminable risks.	Fear of uncertainty, unknown
I tried to avoid risks because I am scared to lose my reputation if I fail. After this experience, I understood that the best way to learn is from own mistakes.	Fear of damage to reputation or ego	Motivation
It happens to me often that I am not able to persuade people to take my desired action. I think a reason for that is my fear to offend people.	Fear of negative impact on others	
If I feel unfamiliar with something or there is a possibility of rejection, I tend to become more introverted and it is difficult to work up the courage to act.	Fear of negative response by others	
Whatever your life looks like, see that there is more and experience it! Take risks and be brave to live this one and only life you will ever have.	Importance of leaving comfort zone	
It worked out and it showed me that I should do this much more often. Too many times limiting beliefs are hindering me to further develop myself.	(idem)	
Because of this whole experience I realized, or rather remembered, that getting out of my comfort zone was actually really beneficial and even made me feel good about myself afterward. It has been some time since I have properly challenged myself.	Satisfying nature of going out of comfort zone	
Until April 5, 2016, I had no idea that being constantly alert to seek opportunities, to persistently take action, and to be out of your comfort zone for 48 h was so tiring. And fun.	(idem) +	
A personal pitfall of mine is seeing risks and challenges in almost everything instead of opportunities.	One's own personality traits –	
First of all, my thoughts about myself can hinder me of doing something and being creative and innovative. Secondly, I might also have some prejudices of other people and only until I can let them go, I am able to seize all of the opportunities that are offered to me.	Biases, prejudices	
The other team members came up with the idea of going to a school and doing a presentation of some sort for the children. I was not comfortable with this idea right from the start, as we didn't seem to have any idea what we were going to do for the children.	Lack of a plan
It was definitely a step out of the comfort zone. Together with my fellow students, I had to convince a stranger to sit down with us, have a coffee and start a discussion. It worked out and it showed me that I should do this much more often.	Interacting with strangers	Capability
We decided to form a choir. However, singing for us was something that we are not really used to, especially in public. So, this task was quite far from our comfort zone.	Doing unfamiliar things	
After being put to a situation where exposure and action were a must, I quickly started trusting my knowledge and skills to complete the task.	Prior experience, skills, knowledge	
We tried to minimize the risk with a good network and attractive performance at our event.	Use of risk management strategies	
I was aware that everything usually does not go as planned but I feel comfortable to have some sort of plan before heading out.	Having a plan +	
I was afraid that my action of introducing these two people would create no value to them, or just to one side, in the worst case making them feel uncomfortable.	Lack of value creation –	Social norms
When I read the assignment paper, I remember thinking “oh boy, this is going to be a big challenge. It's not normal for Finns to try to engage people they don't know at all.	Violating societal norm	
For me, it was easiest to take action when I knew I would be adding value to someone's life or providing them with something.	Truly creating value	
I was more courageous than usual because I was part of a team, and this helped me to behave in a more resourceful way.	Not to disappoint the team +	
I was feeling desperate. Standing outside of the classroom with three other people I had just been introduced to a few days ago, trying to figure out how to complete a series of tasks, which—in all honesty—seemed completely impossible to accomplish.	Seeming impossibility of – meeting the challenges	
I was assuming that they will not be receptive when I approach them. But I later realized it was my attitude and lack of courage that affected my decision to act or not act at the moment.	Language barriers	
At the meeting, I felt very uncomfortable because it seemed that the woman had not much time and she looked a bit annoyed from the situation.	Actual negative responses from others
Unfortunately, my teammates were not always giving my support which also decreased my risk taking level.	Team difficulties	Course Specific Features and Experiences
We didn't have much time thinking about pluses and minuses of our actions, that is why taking the risk was the only opportunity to understand if we made a right choice or not.	Time pressure
We continuously encouraged each other to take action.	Team support
After successfully completing a few challenges, my confidence grew in doing more things outside of my comfort zone.	Early successes +

Regarding motivation, students mention a range of fears (of failure, of uncertainty and the unknown, of damage to one's reputation or ego, of negative impact on others, and of negative response by others). The learning format is designed to trigger such fears because gaining experience in dealing with them helps to develop entrepreneurial competencies and an entrepreneurial mindset. The listed “+” factors show that what helps in terms of motivation is being convinced of the importance of leaving one's comfort zone. What also helps is the satisfaction that can arise from overcoming such fears by going out of one's comfort zone.

Regarding capabilities, some participants mentioned that they feel limited by their own personality traits and by their biases and prejudices. They also mention difficulties in doing unfamiliar things and interacting with strangers, particularly when having to do this spontaneously or improvisationally, without having a plan. Having some sort of plan can help to venture out of their comfort zone, although one of the mentioned learnings in Table 3 (to be discussed in the next section) is that some participants realized that they can solve problems on-the-go and that a plan is not always needed. Further analyses of the “+” factors show that participants venture out of their comfort zone when they realize that they do have means available (experience, knowledge, skills, and networks) and when they can employ strategies to cope with uncertainty and possible rejection or failure.

**Table 3:** Learning outside the comfort zone.

**Representative quotations**	**First order codes**	**Themes**
I now realize the importance of challenging myself because I am my first opponent, and after taking on one challenge every other will be easier than I previously thought. I also feel that I have what it takes to be successful, it's up to me to take action and work hard in order to achieve it. Looking back on Opportunity day I must say that I was lucky for taking part on it, because it pushed me out of my comfort zone, something that I will have to do more often in the next couple of years.	Importance of leaving comfort zone	Taking action
Because of this whole experience I realized, or rather remembered, that getting out of my comfort zone was actually really beneficial and even made me feel good about myself afterward. It has been some time since I have properly challenged myself.		
Enough talking about how important enterprising behavior is—just go and do it. And I am happy I could be a part of it myself.	Importance of taking action	
I learned that you don't necessarily have to plan everything and that it is better to face your fears than to try and evade situations that make you uncomfortable.		
I realized that many things look more complex than they actually are. You only have to dare to start and normally the next step arises from the one ahead of it. It is not beneficial to get into detail when you plan something. It is too risky to get loose track which might lead to the fact that you have to change your plan a thousand of times till the end.		
Gets people to actually do something (a lot!) AND they happily do more than requested!	Effectiveness of the format	
When I signed up for this course, I expected that it would be great, but what I did not know is that it would change my perspective and that it made me think about topics which I do normally not consider. This course made me so enthusiastic and showed me that the impossible can become truth.		
I was initially cynical as to whether or not it's at all possible do anything at all as such an *ad-hoc* agent. No, even'cynical' is too weak of a word. I was convinced that such an avenue of action wouldn't lead anywhere. Shows me what I know, right? Clearly, by doing and trying things you can not only learn stuff, your perception of what's possible and what will not change and your courage to do things will be enhanced. Which in turn will quite likely lead you to discover and try even more possibilities.		
To sum up, I have learned a lot on the Entrepreneurial Behavior Days. Normally courses are held in class and a professor is teaching frontal. However, in this course, we had to prove ourselves and tried to best organize the short time of < 48 h. I learned more about my soft skills and was even able to provoke myself in terms of new challenges, I never did before.		
It is very meaningful for me to be aware that I definitely can find the opportunities in various ways and also can transform the ideas into action if I surpass my own fear and being confident. I hope I can keep this spirit in the long run to gain success.	Increased confidence in own capability	Competence
Looking back, I would say that this opportunity day was a good experience for me, and I learned a lot: I just had to do thing that aren't in my comfort zone. I can achieve a lot if I just put my mind into it. I believe I have discovered new things about what I am capable of, and it is up to me now to put these competencies to good use.		
It was amazing to see how many things you can reach and make possible in 48 h.	Enhanced sense of agency	
The day was full of challenges and opportunities to be found and utilized. The day was amazing. When I woke up on that Wednesday morning, I could have never ever thought that we could do this all.		
For me, the Action Learning Day was an excellent experience and not something that I would have done by myself. My key take away is that engaging in enterprising behaviors is not a risky as I had previously through—the worst that someone will probably say is “no, go away,” which is not really that bad (and if nothing else you have just learned how not to approach someone in a particular way).		
There's a personal, contributing factor which has only recently started to crumble: the perception of the world as something immutable, or at least immutable for mere mortals. Only disturbingly recently has the fact dawned on me that everything's actually made by normal people, the people you meet on the street.		
I have observed I am risk-averse and very much like to stick to the plans (apart from when I am delaying assignment reports). I believe it is amazing what could be achieved if people dared more, me inclusive.	Awareness of internal constraints	
In short, my prior knowledge and attitudes initially gave me an extremely strong negative feeling about this assignment. Needless to say, I immediately recognized that it was the completely wrong attitude to have. Between my undergraduate and graduate education, I have never had the opportunity to go out and practice the exact theories I had learned. I quickly realized that this was a great opportunity to do so.		
I learned that asking someone to do something is not a problem and that you don't have to ask yourself any question about it and your legitimacy to do so as long as you make sure that it creates value for him, which I think is also a great lesson.	Importance of truly creating value	Creating value
I said that if we can create some values for them, it is not going to be awkward (…) What I have learned is how important creating a value for people is. Because if people are given something good, they feel happy. This is really simple and basic thing but important.		
I refrained from action because I only saw myself as taking value and therefore feared a negative reply. It did not come to my mind that the officer would be happily surprised by our question and that he enjoyed this spontaneous action.		
In these days, I have learned a lot about myself. The most interesting part for me was that I realized that there are so many ways you can create something and to make a value to society. Everyone can contribute a lot.	Opportunities to create value in daily life are all around	
To sum up, what I have learned is that we can see various opportunities every time, everywhere. Important thing is to see usual day/regular life as different perspective. Because there are so many things we don't usually pay attention to. However, thanks to a change in perspective, we can now notice new things, even if those things can be seen as normal things by others. And combine our resource/capability with opportunity we see.		
I was really surprised how powerful and useful networking is. The most difficult challenges we only could accomplish because of our network contacts. I realized that my network is not big but diverse. So, I always found a right contact to get things done.	Power of the team and of networks	Working with others
My most significant learning concerning networking and reciprocity concerned the relationship between trust and liking when leveraging networks in a high-pressure situation such as an EB Day. I usually establish networks based on mutual liking, but in engagements which take less than a minute in some cases, the establishment of trust is more important. When engaging with people and asking favors, it was far more effective to make people understand that cooperation with the team was a safe thing to do, rather than it being important that the person liked me first. In short, when there's no time for charm, go for trust.		
One of the first things that we learned is to exploit the capabilities of each one. Although we did not know previously, we soon realized to in what field each one was good and, thus, each played a “role” inside the group to work in the most effective manner possible.		
I am going to spend more time focusing on the social aspect of the tasks and challenges that I am presented at work and school. In my work everything at first glance appears to be task focused, as results are key, however, the social focus gets overlooked as I have the comfort of my network and my communication in English. Being stretched to accomplish these challenges without my network and language barriers really highlighted the importance of the social aspect of a task and how it alone can lead to success.		

The third theme concerns social norms. Participants feel the inability to leave their comfort zone if they feel that what they attempt to provide offers insufficient value. They also feel hesitant to take action and create value if the required actions appear to violate societal norms, for example, approaching strangers. The first factor under “+” provides an antidote: to truly create value. If the ideas for created value are believed in, then this helps to overcome the pressures of the social norm-related factors to stay within the comfort zone. This is further helped by doing the challenges as a team. Participants mentioned that they act more courageously than they would normally do, as they want to pull their weight in the team and do not want to disappoint their team members.

The last theme refers to attributes and experiences specific to the learning format. The first factor mentioned under “–” is that the challenges initially overwhelm participants, also in relation to having very limited time to work on them. However, some initial successes, even if small, help teams to gain confidence, encouraging them to take further actions to create value. Still, teams will invariably also receive negative responses from people they approach, which may further condition them to stay in their comfort zone. Time pressure, although initially contributing to the impression that the challenges are formidable, typically acts as a strong incentive to venture out of their comfort zone. Students do not want to fail the assignment and the time pressure creates the urgency to act, up to the extent that there is even too little time to complain to the instructor about a lack of time. Team difficulties serve as a further limiting factor, with team support as its opposite. The task, therefore, is for team members to engage in attempts to improve communication and the psychological climate in the team. Techniques to do so can be frontloaded (see Description of the Learning Format section). As with any competency, such frontloading affects all other competencies: with better teamwork, members are more willing and able to share ideas, take action, open their network, convince people, and persevere.

The factors that act as pressures to stay in the comfort zone (marked “–” in Table 2) indicate the relevance of the exercise. As emphasized by Daspit et al. (2021)'s definition, those with an entrepreneurial mindset can create value under conditions of uncertainty. Thus, the possibility of failure is imperative when practicing taking action (taking risks and overcoming fear) and perseverance. As such, the factors listed by the participants act as pressures to stay inside the comfort zone and double as design principles for the challenges. When using a comfort zone model pedagogy to stimulate an entrepreneurial mindset, the challenges provided to participants must be designed in such a way that participants will feel uncomfortable. Without such limiting factors present, it would not be possible to employ a comfort zone model of learning.

### The learning zone: changed beliefs and expansion of the comfort zone

The second empirical research objective is to establish the types of realizations that participants have. The thematic analyses reveal four themes around which learning occurs (see Table 3): realizations pertaining to taking action, one's own competence, the creation of value, and the role of “others.” Generally, learning is associated with surprise: participants come to realize things that they did not know before or had not expected. To facilitate surprise learning, the unexpected and secret character of the challenges is greatly helpful if not imperative. The challenges invite novel behaviors which are helpful to shake up habits and assumptions. Typically, many unexpected things and events happen during the action learning days. Successes are important, but they only lead to learning if they have an unexpected character. Easy and safe successes bring little learning and as such little development of entrepreneurial competencies and mindset, and are therefore discouraged in the learning format instruction (see Supplementary material). Given the association of learning with surprise (cf. Luna and Renninger, 2015), surprise has been used over the years as a criterion to select and develop challenges. If reflections would refer to challenges in connection to surprise learning, they would be retained, and if not, they would be removed.

The first theme (Table 3) concerns surprises and realizations pertaining to taking action. Subthemes are the importance of leaving the comfort zone, the importance of taking action, and the effectiveness of the learning format for competency and mindset development. Seeing the importance of taking action and of leaving their comfort zone is not only a motivator propelling students into action (Table 2) but it can also be a result of engaging in the format. Students report that engaging in entrepreneurial behavior in this fashion can not only result in personal development but can also be satisfying as well as fun. Several students report that the effectiveness of the format surprised them, sometimes overcoming initial skeptical beliefs.

A second set of learnings revolves around one's competence. The quotes shown in Table 3 express an increased confidence in capabilities, and an enhanced sense of agency. One example of a changed capability belief is that one can step out of their comfort zone and go through barriers. Another one is that the pain of being rejected often quickly reduces so one can become less vulnerable by being vulnerable. Another realization concerns the ability to let go of control and trust one's ability to think on one's feet and to make things happen on the go. This can be an eye-opener for university students as the cognitive character of traditional formats conditions them to plan and prepare, often without ever getting into action. Some students also report that they became more aware of internal constraints holding them back from taking entrepreneurial actions. This can be used in later modules, asking students to work on specific competency aspects.

A third realization concerns the importance of creating value. Truly creating value not only puts other people in their comfort zone but also helps the participants to believe in what they are doing. Believing that what you offer is beneficial to others, enables action. Even better is that opportunities to create value for others are plentiful. Students also report that because of taking part, they started to notice that opportunities to create value in daily life are ubiquitous. Every day, there are limitless opportunities to connect, help, share, show interest, organize, invite, etc.

A set of final realizations concerns the role of “others” in effective entrepreneurial behavior: the importance of one's teammates and networks for success. Even strangers are often found to be much more cooperative than initially assumed. Students are also able to experience first-hand the power of having a good network and the power of having a good team. They report the realization that contributing to positive social processes in the team helps all members to take risks, grow, and learn.

## Discussion

Hultén and Tumunbayarova (2020, p. 9) asked and answered the following question: “So, what should we do differently in entrepreneurship education to develop the students' entrepreneurial mindset? If this is our intention, then it is not enough to rely solely on pedagogical methods that develop the student's knowledge and skills. Thus, it is necessary to include pedagogical interventions that enhance the students' beliefs in their own capabilities to respond creatively to opportunities despite obstacles and uncertainties. The instructor needs to use exercises that engage the students and even make them forget that they are students. Creating conditions for collective group learning is important and we have found that playful exercises that challenge the participants' creativity and force them to think and act “outside the box” are means to this end. These exercises make the students leave their mental comfort zone and take steps beyond their usual thinking and acting, which later make it possible for them to reflect over[sic] their thoughts and actions. Thus, doing something out of the ordinary is critical for the development of a learning climate of play, experimentation, [sic]trial and error, which make it possible for the students to see themselves and their peers from a new perspective.” The learning format presented in this article complies with the suggested characteristics.

The analysis in the Findings section reveals that participants learn when they have realizations that surprise them. Students report instances of changes in beliefs and perceptions indicative of transformational learning (Mezirow, 1997). The more that students enter the learning zone and grow their comfort zone for entrepreneurial behavior, the more successful the learning format is. Thus, it is important to know the factors that contribute to having significant learning experiences. There is no guarantee that these learning experiences will occur. The effectiveness of the learning format is not just determined by the format's features, but at least equally by the extent to which participants take the risk and venture out of their comfort zone. Furthermore, if learning arises, they are not uniform. Each participant's journey is different because of differences in interpretations of the challenges, ideas for opportunities, actions taken, and responses encountered. Additionally, the format addresses soft skills, not hard skills, which means that internal standards for assessment shift as part of the experience. Participants could in principle score their competency level similarly or even lower after making progress, having discovered some limitations they were previously unaware of, even if they worked on overcoming these limitations. For all these reasons, participants' reflections give better insight into the impact of the learning format than competency proficiency scales would do. Because of the wide variety of experiences and lessons learned, numerical evidence for the effectiveness of the learning format is best provided by aggregate ratings of the course as provided by regular university evaluation systems. The overall evaluation scores were 4.5/5 in Russia, 4.6/5 in the Netherlands, and 4.9/6 and 5.8/6 in Austria, in the editions from which the reflection reports were selected. In Russia and the Netherlands, the learning format was a module in a course. For the other three locations, no evaluation scores were available.

Team members who do not apply themselves tend to learn little (although sometimes they learn that they missed an exciting opportunity to learn, after seeing the accomplishments of other teams or team members during the action learning days). To a certain extent, it is unpredictable what will happen during the presentations following the action learning days. However, it is important to provide autonomy to the students, so that they can be self-starting and design activities that are matched to their competency level and their comfort zones. This allows them to engage in self-directed learning (Lindberg et al., 2017a,b). The absence of the teacher during the fieldwork is a powerful ingredient in this regard. The open-ended nature of the challenges also helps to tie actions to individual team members' comfort zones. Some may be taking small steps while others act more boldly. However, what matters is whether a participant expands their comfort zone. Furthermore, the dynamics of the teams cannot be controlled. By providing participants with a short training beforehand on the basics of teamwork, in particular, the management of social processes within the team, the program can be designed to optimize the chances that significant learning benefits will occur and that the participants' comfort zone will expand.

This study raises some questions which may be directions for future research, and the lack of current answers may be seen as a limitation of this study. First, coming back to the distinction between enterprising and entrepreneurial made in the “Entrepreneurship, Competencies, and Mindset” section, in what ways is enterprising behavior in daily life relevant to entrepreneurial behavior in the sense of starting or running a business? The enterprising behavior format discussed in this article is mostly concerned with value creation, not so much with value appropriation. With venture-directed entrepreneurship, value appropriation is also important, if the venture is to be viable. A related difference concerns the role of risk. Engaging with risks and the possibility of failure are good things for learning enterprising behavior and developing an enterprising mindset. In contrast, in venture creation, taking risks is essential but at the same time, risks are to be managed and reduced. Relatedly, the feasibility of actions is not conducive to the expansion of the comfort zone, and participants are discouraged to take safe actions merely to “tick the box.” In contrast, in venture creation, feasibility may be conducive to making a profit. One answer to the question posed at the beginning of this paragraph is that it is difficult to envisage successful venture-directed entrepreneurial behavior without possessing the enterprising competencies practiced in this format. Thus, the question becomes whether the learnings transfer to other situations: whether participants will act in a more enterprising way in later and different situations, now that certain elements of the course will not be present (e.g., the team, the course credits, and the university environment).

A second issue concerns the differences in learning experience per country. The analyses involved participants from five countries (Austria, Finland, Germany, Holland, and Russia). First, participants learn more if they are diligent and willing to apply themselves, and this may partly reflect national education systems. Second, in terms of countries, practicing entrepreneurial behavior using these formats is harder if a culture has low levels of trust and if a culture is rather hierarchical and bureaucratic. On the other hand, in those cultures, highly unexpected experiences can occur, which makes learning even more pronounced. Cultures also differ in terms of how free the students feel to express themselves—the freer, the better for entrepreneurial behavior. In sum, the development of entrepreneurial competencies and an entrepreneurial mindset is harder in some cultures, but in such cultures, there is also more potential gain. This mirrors the findings for the effectiveness of entrepreneurship education more generally (Bae et al., 2014; Walter and Block, 2016; Lyons and Zhang, 2018).

The learning format described in this article provides a flavor of entrepreneurial behavior without doing anything business-level or venture related. It is therefore suitable for students who do not yet have their own business, have plans to start such a business, or have business experience or even work experience. It allows them to engage in entrepreneurial behavior in daily life and build their confidence in doing so. Because daily life offers ample opportunities for value creation, the learning format can readily be applied in any place at any time. The analysis reveals that the exercise can produce changes in beliefs and perceptions. As such, this study contributes to the body of knowledge on interventions to develop individuals' entrepreneurial competencies and mindsets in non-business settings.

## Data availability statement

The datasets presented in this article are not readily available for privacy reasons. Requests for access to anonymized data should be directed to m.w.van.gelderen@vu.nl.

## Ethics statement

Ethical review and approval was not required for the study on human participants in accordance with the local legislation and institutional requirements. Written informed consent for participation was not required for this study in accordance with the national legislation and the institutional requirements.

## Author contributions

The author confirms being the sole contributor of this work and has approved it for publication.

## Footnotes

^1^Edwin de Bree originally came up with the idea for this learning format. Between 2009 and 2012, I developed the format into its final form. Since then, it has been tried and tested in 24 courses, with 721 participants, at six universities in five countries (Austria, Finland, Germany, Holland, and Russia) between 2013 and 2019. COVID-19 then paused its use, as the format requires interactions with strangers.

^2^For these reasons, the challenges are not provided in this article, apart from the one just mentioned. Instead, they will be provided by author upon request (m.w.van.gelderen@vu.nl).

^3^My publicly available website (www.enterprisingcompetencies.com) contains overview of articles I authored.
