# Supplementary material for: Using a comfort zone model and daily life situations to develop entrepreneurial competencies and an entrepreneurial mindset
Source: Front Psychol. 2023 May 15;14:1136707. doi: 10.3389/fpsyg.2023.1136707 (PMC10225726; doi:10.3389/fpsyg.2023.1136707)
Supplement: Supplementary file 1 [file Data_Sheet_1.pdf]

Appendix A: Course/Module Guide Enterprising Behavior

[Coordinator / Lecturer / Instructor]

[Provide contact details here]

Lectures and activities:

Day 1	Time	Lecture – Introduction to Competencies; Generating Ideas for Opportunities; Teamwork
Day 2	Time	Lecture - Networking; Taking Action; Perseverance; Convincing Others
Day 3	Time	Enterprising Behavior Day 1<br>(action learning)
Day 4	Time	Enterprising Behavior Day 2<br>(action learning)
Day 5	Time	Presentations<br>Wrap up
One week later	Submit assessment by [time] at the latest	

Contents

This [course/module] involves the study and practice of enterprising behavior. Six competencies are discussed and practiced in class: generating ideas for opportunities, taking action, perseverance, networking, teamwork, and convincing others. The level of analysis in this part of the course is the individual: we will not be discussing enterprising firms, but enterprising people. Enterprising behavior can be expressed in starting a new venture but also in a multitude of other endeavors. You will not start a business in this course but you will be invited to take a lot of enterprising action during the Enterprising Behavior Days.

While the unit of analysis in this course is the individual (mostly you), the competencies that are studied and practiced are highly relevant in the case that you will start or run your own venture. Even if you are an expert in finance, logistics, marketing, production, organization, etc., you will not get far if you are unable to generate ideas for opportunities, take action, persevere, network, work in a team, and convince people. Moreover, these competencies are not just important for business ventures, they are also increasingly relevant in the wider societal context. Nowadays there is a huge expectation of self-reliance. Governments and

companies are less willing to support and help you. In this context, you are increasingly dependent on well-honed enterprising skills.

Learning outcomes

Learning outcomes describe what you should know and be able to do by the end of the Course

- Learning outcome 1: To have acquired knowledge with regard to enterprising competencies
- Learning outcome 2: To have developed insight into, and actual development of your own enterprising competencies

Assignment

The Enterprising Behavior Days will offer an exciting trip that will take teams of participants around [name of city] and beyond for [one/two] full days. During this trip teams will work on a variety of challenges that are all designed to allow you to show enterprising behavior. The development of enterprising skills is taken here in a broad sense – not necessarily related to starting a new venture.

The competencies that are practiced during the Enterprising Behavior Days are:

- Generating ideas for opportunities, creativity, innovative behaviour
- Taking action, risk taking, guts, daring, assertiveness
- Perseverance
- Network and resource utilization, improvisation
- Teamwork
- Convincing others, Persuasion, engaging people, influence

Combined, they will help you to create value for other people.

During the Enterprising Behavior Days you will work in a team; however the assessment is individual.

The teams will be formed on the second day of the course. Teams will consist of four persons each. You will need to take part for the entire two days, and attend the presentations the next day. Cancel any other obligations you may have. Participation is mandatory.

The challenges will be handed out on the morning of the **3rd day**, at [time] sharp. The place will be announced. You are expected to work on the challenges for the entire two days. Bring your I.D., public transport card, mobile phone, and some money. A car can be handy but not necessarily. You do not need to connect beforehand with your team. You cannot prepare for

the Enterprising Behavior Days. However, you need each other's contact details, which will be provided.

Try to fulfill the challenges **in the most enterprising way possible** (rather than doing things quick and easy just to "tick off" another challenge). Make sure that you collect proof (pictures, movie clips, business cards and such). When using your camera, ask for permission. Be polite at all times – you are representing [name of University/Institution]. Stay together as a team, do not split up.

The Enterprising Behavior Days have a competitive element: You compete with the other teams for the title of "Most Enterprising Team". The winner is chosen during the presentation session on the **5th day**. The lecturer and the other teams will decide on the winner (teams cannot vote for themselves). The choice for the winner is decided right after the presentations.

During the presentations on the **5th day** each team will present what they have done during the Enterprising Behavior Days. The purpose of the presentation is to convince the audience that your team is the most enterprising team. In your presentation you should use the evidence that you have gathered during the days: pictures, movie clips, business cards etc. Make clear for every challenge how enterprising your team has been. Obviously, more enterprising teams can make more credible claims that they deserve to be the winner.

The Enterprising Behavior Days provide you with a unique opportunity to design your own learning experience. Own interpretations, original approaches, or even partial or full replacements of challenges by ones that you are coming up with yourself are highly appreciated, **if** you are able to demonstrate convincingly that these new challenges helped you to show enterprising behavior even better.

Teams stick together during the Enterprising Behavior Days! Do not split up. Make sure that you have a lot of fun, and come back with some good stories.

Assessment

On **one week after the 5th day**, by 23.59pm, you will submit a reflection on your behavior during the Enterprising Behavior Days. Please send to [email coordinator].

Keep close track of what do you during the Enterprising Behavior Days, and also of feedback that you might be given during the presentations on the **5th day**. This will help you to write your reflection.

In the assignment, analyze your own behavior per set of competencies listed above ((1) Generating Ideas for Opportunities; (2) Taking Action; (3) Perseverance; (4) Networking and Network Utilization; (5) Teamwork; (6) Convincing Others. Use the competencies as headings. Briefly describe what happened during the challenges and focus your analysis on your own behavior. Be specific in your self-analysis: analyse why behave as you did, and

anlyse any situational and personal influences on your behaviour. Also analyze what aspects can be improved, and make a plan for how you will improve your enterprising competencies.

You are not only asked to reflect on your behavior. You are required to incorporate constructs and theories from the prescribed readings in your reflections. You have to show your ability to apply the literature to your experiences, and your experiences to the literature. You can treat the literature critically. Not referring to constructs and theories from the prescribed readings will result in a poor grade.

Spend far more words analyzing your behavior than describing your behavior. Do not submit an assignment that merely consists of descriptive material (e.g., "I first did this, and then we went there, and then we did that, and I said so and so"). Write in the "I" form as much as possible (rather than the "we" form).

The assignment is not meant to convince the lecturer that your enterprising skills are well developed! It is not meant as proof that you were very enterprising. Rather, it should show that you are able to analyze your own behavior, and that you are able to think of ways to improve. The purpose of the assignment is to give insight in your own enterprising skills and their development. That said, the more enterprising you were during the Enterprising Behavior Days, the more you can reflect on. Therefore, effort during the Enterprising Behavior Days is an important assessment criterion.

Use the following structure: title page, introduction, discussion (use the competencies as headings), plan for development. The word count (1750 words +/- 10%) concerns the entire content of the report.

Marking Schedule for Assignment

Part	Explication
Introduction<br>(weight: 5%)	Start with a few interesting sentences that entice the reader to read further. Clear and short explanation of the topic and the purpose of the report. Brief overview of what the reader can expect
Effort and display of enterprising skills<br>(weight: 40%)	Amount of effort made. Amount of enterprising behavior shown during the Challenge. Degree of challenge, risk and novelty.
Reflection on skills and application of the literature (weight: 40%)	Your behavior is briefly described and then analyzed in terms of the enterprising skills studied and practiced in the course. Depth of application of the literature. Keeping your own 'voice'.

Plan for development (weight: 10%)	Number, depth, and specificity of suggestions for the future development of skills. Use of implementation intentions.
Presentation (weight: 5%)	Attractive title page, quality of writing, grammar, and spelling, lay-out of the report, use of Arial or Times New Roman, 11-point font, 1.5 line spacing, APA referencing style, clarity of structure, use of headings, use of color, inclusion of images and pictures

You need a [minimum score of xx] to pass the [course/module].

Revisions are [allowed / not allowed].

For revisions, a grade cap of [xx] applies.

Readings

Readings can all be downloaded from www.enterprisingcompetencies.com

Enjoy the Course/Module!
